# CRONE PEDAGOGIES: EMANCIPATORY PRAXIS

**DOI:** 10.1093/geroni/igad104.2026

**Published:** 2023-12-21

**Authors:** Nicholas DiCarlo

**Affiliations:** UCSF / Hunter College / Routledge, New York City, New York, United States

## Abstract

Confronting ableism and ageism in gerontological spaces promotes an intergenerational praxis rooted in an ethics of care. “Crone pedagogies” signal teaching and learning that raises consciousness collectively across generations by confronting the coloniality of everyday life and challenging the ways in which people are forced to cope with unjust systems (Philips, Adams, & Salter, 2015). This framework extends from the critical gerontological work and mentorship of Carroll Estes. The emancipatory potential flourishes from the restoration of historical memory (Martín-Baró, 1994) and the futurity that intergenerational work embodies. This presentation features examples from teaching practice and scholarship, within and outside the academy, while encouraging real-time experiential learning through reflective awareness about intergenerational access within the conference space.

